# Circulating Tfh cell and subsets distribution are associated with low‐responsiveness to hepatitis B vaccination

**DOI:** 10.1186/s10020-021-00290-7

**Published:** 2021-04-01

**Authors:** Mingjuan Yin, Yongzhen Xiong, Dongmei Liang, Hao Tang, Qian Hong, Gang Liu, Jinmei Zeng, Tingyu Lian, Jingxiao Huang, Jindong Ni

**Affiliations:** 1 Department of Preventive Medicine, Guangdong Medical University, Dongguan, China; 2 School Clinic, Guangdong Medical University, Dongguan, China; 3 Department of Epidemiology and Biostatistics, Guangdong Medical University, No.1 Xincheng Road, 523808 Dongguan, China; 4 Teaching&Research Department, Dongguan Guancheng Hospital, Dongguan, China; 5 Dongguan Guancheng Hospital, Dongguan, China; 6 Department of Immunization Program, Shenzhen Center for Disease Control and Prevention, Shenzhen, China

**Keywords:** Circulating Tfh cells, Low‐responsiveness, miR-19b-1, miR-92a-1, Hepatitis B vaccination

## Abstract

**Background:**

An estimated 5–10 % of healthy vaccinees lack adequate antibody response following receipt of a standard three-dose hepatitis B vaccination regimen. The cellular mechanisms responsible for poor immunological responses to hepatitis B vaccine have not been fully elucidated to date.

**Methods:**

There were 61 low responders and 56 hyper responders involved in our study. Peripheral blood samples were mainly collected at D7, D14 and D28 after revaccinated with a further dose of 20 µg of recombinant hepatitis B vaccine.

**Results:**

We found low responders to the hepatitis B vaccine presented lower frequencies of circulating follicular helper T (cTfh) cells, plasmablasts and a profound skewing away from cTfh2 and cTfh17 cells both toward cTfh1 cells. Importantly, the skewing of Tfh cell subsets correlated with IL-21 and protective antibody titers. Based on the key role of microRNAs involved in Tfh cell differentiation, we revealed miR-19b-1 and miR-92a-1 correlated with the cTfh cell subsets distribution and antibody production.

**Conclusions:**

Our findings highlighted a decrease in cTfh cells and specific subset skewing contribute to reduced antibody responses in low responders.

**Supplementary Information:**

The online version contains supplementary material available at 10.1186/s10020-021-00290-7.

## Introduction

There are 3.5 % of the global people chronically infected with hepatitis B virus (HBV), ultimately resulting in cirrhosis complications and hepatic carcinoma (Hutin et al. 2018; Yuen et al. 2018). As hepatitis B vaccination has been widely available across the world over recent decades, incidence of HBV infections is decreasing. However, epidemiological studies have demonstrated that an estimated 5–10 % of healthy adults lack adequate antibody response following receipt of a standard three-dose hepatitis B vaccination regimen (Zuckerman 2006),which leave people susceptible to the threat from HBV infection. In this regard, ascertaining the immunological events that lead to poor responses to hepatitis B vaccination will be of great value in controlling HBV infection, especially in susceptible adult populations.

The cellular mechanisms responsible for poor immunological responses to hepatitis B vaccine have not been fully elucidated to date. For many decades, T follicular helper (Th)1 and Th2 cells have been considered to predominantly regulate the poor immune response to hepatitis B vaccination, but still with controversial results (Larsen et al. 2000; Wataya et al. 2001). Emerging evidence suggests that T follicular helper (Tfh) cell-the major helper T cell subset are instrumental in humoral adaptive immune response. Tfh cell which characterized by high levels of CXCR5 have gradually gained recognition in the formation of germinal centers and specialized in facilitating B cell affinity maturation and antibody class-switching (Vinuesa et al. 2016). Growing evidence showed that Tfh cells can be divided into three subpopulations: Tfh1 (CXCR3^+^CCR6^˗^), Tfh2 (CXCR3^˗^CCR6^˗^) and Tfh17 (CXCR3^˗^CCR6^+^) cells according to the expression of CXCR3 and CCR6 (Morita et al. 2011). Among this, the CXCR3^−^subsets of cTfh cells were main functional subsets, which can induce differentiation of naive B cells into antibody-secreting cells (Ueno 2019; Song and Craft 2019). Nowadays, circulating Tfh cell (cTfh) is well recognized as a substitution of lymph Tfh cell cells (Asrir et al. 2017)and cTfh has been reported in vaccines, infectious diseases and other human immune system disease (Crotty 2019). However, little is known about the part of Tfh cells and subsets in low-responsiveness to hepatitis B vaccination.

Furthermore, recently identified microRNAs may regulate Tfh cell differentiation and function, or even play a dual role in manipulating Tfh cell differentiation depending on the time or infection specificity (Lee et al. 2018; Ichimiya et al. 2017). While we previously reported initial studies exploring dynamic changes in Tfh cells and crucial microRNAs after revaccination with hepatitis B vaccination (Xu et al. 2018), the role of Tfh cells miRNA regulatory mechanisms in the poor response against HBV remains unclear. In this regard, we identify specific changes in Tfh cells subsets and microRNA that were related to low hepatitis B vaccine-induced antibody generation in the current study.

## Patients and methods

### Low responders

**Fig. 1:**
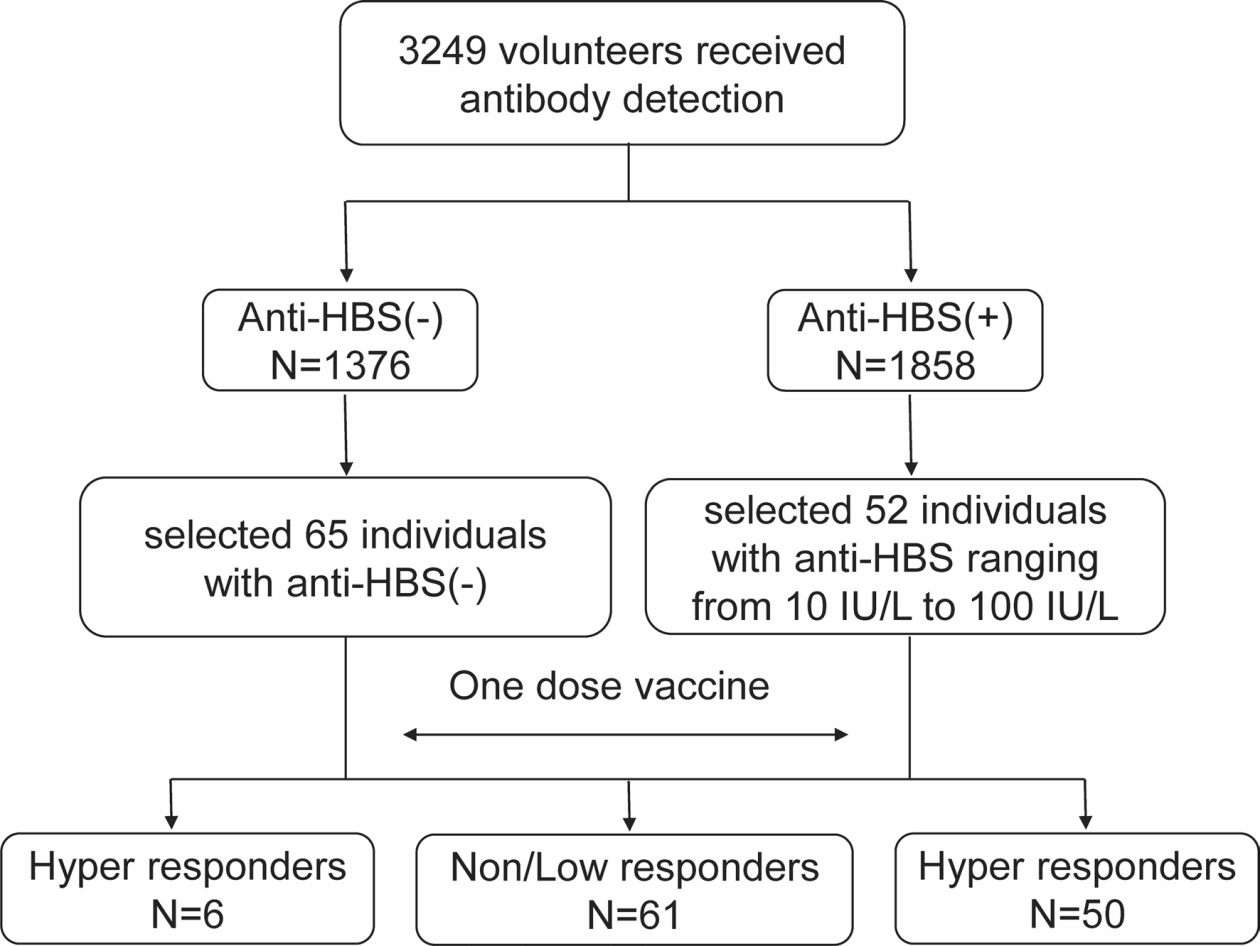
Flow diagram for low responders and hyper responders recruited in our study

Details of the screening for low responders (LR) are described in Fig. 1. In brief, a total of 3249 freshmen with hepatitis B vaccine history underwent serum anti-HBs detection during a physical examination following school enrollment at Guangdong Medical University in the fall of 2017 (In China, hepatitis B vaccine was included into Immunization Program management in 1992 and then into the National Immunization Program in 2002. This situation provided a good way to find a non/low responders). In addition, 65 volunteers from 1376 anti-HBs-negative volunteers and 52 volunteers from 1858 anti-HBs-positive volunteers (with anti-HBs-positive titers ranging from 10 IU/L to 100 IU/L) that complied with the strict inclusion criteria were enrolled in further experiments. If anti-HBs-negative recruited individuals have been infected HBV, they also could not induce antibody production after vaccination. We performed detection of five tests related to HBV including HBsAg, hepatitis B e antigen, anti-HBs, antibodies against HBeAg and antibodies against hepatitis B c antigen in anti-HBs-negative recruited individuals in order to exclude infected people mixed in low responders. The 65 eligible volunteers were revaccinated with a further dose of 20 µg of recombinant hepatitis B vaccine (Saccharomyces cerevisiae) (Shenzhen Kangtai Biological Products Co., Ltd., Shenzhen, Guangdong) which can identify primary immune failure and secondary immune failure. Peripheral blood samples were collected at D7 (range 7–8 days after vaccination) and D28 (range 28–29 days after vaccination) after vaccination and part samples were collected at D14 (range 14–15 days after vaccination). The anti-HBs titers were typically assessed approximately one month after vaccination. Thus, we assessed vaccine responses at D28 after revaccination and different vaccination efficacy groups in our study were defined as follows: anti-HBs-negative and anti-HBs-positive titers ranging from 0 IU/L to 100 IU/L were regarded as LR, whereas anti-HBs-positive and anti-HBs titers greater than 100 IU/L were regarded as hyper responders (HR). Ultimately, there were 61 LR and 56 HR involved in our study. Demographic information is summarized in Table 1 and differences were not statistically significant between LR and HR with regard to age and sex.

**Table 1.** The demographic charateristics of subjects

Parameters	Low responder	Hyper responder	*P* value
Number	61	56	
Age (years)			
Mean±SD (range)	19.3±1.0 (18-24)	19.46±1.49 (17-25)	0.470
Sex N(%)			
Male	18(29.5%)	19(33.9%)	0.607
Female	43(70.5%)	37(66.1%)	

This study was approved by the Ethics Committee of the Affiliated Hospital of Guangdong Medical University (Permit Number: PJ2015034KT).Written informed consent was provided by individual participants following a clear explanation of the study process, benefits and possible adverse effects.

### Multicolor flow cytometry

Similar methods of processing of blood samples and flow cytometry have been reported by our previous study (Xu et al. 2018). Surface staining with antibodies for T cell staining was Anti-human CD4 PerCP-Cyanine5.5 (eBioscience), Anti-human CD185(CXCR5) PE (eBioscience), Anti-human CD183(CXCR3) PE-Cyanine7 (eBioscience), Hu CD196 (CCR6) BV421 11A9 (BD Biosciences) and B cell staining was Hu CD19 PE HIB19, Hu CD27 FITC M-T271 and Hu CD38 BV421 HIT2 from BD Biosciences company.

### Determination of serum anti-HBs and IL-21 by ELISA

Guangdong Provincial Institute of Biological Products and Materia Medica in China performed anti-HBs detection of a physical examination using an ELISA kit (WANTAI BioPharm, Beijing, China). The levels of anti-HBs and IL-21 were also quantitatively determined post-vaccination using the Human HBsAb ELISA kit and the Human IL-21 ELISA kit, respectively (Da An Gene Co. Ltd., Guangzhou, China) according to the instructions provided by the manufacturer and all standards were tested in duplicate.

### Cell isolation and real‐time quantitative PCR

Similar methods have been reported by our previous study (Xu et al. 2018). In brief, cDNA was generated with the Transcriptor First Strand cDNA Synthesis kit (Roche, Mannheim, Germany), and qRT-PCR and the analysis of mature miRNAs was performed using the FastStart Universal SYBR Green Master kit (Roche) and the All-in-One miRNA qRT-PCR Detection kit (GeneCopoeia), respectively, on an ABI 7500 PCR instrument (Applied Biosystems) in a total volume of 10 µL. The optimum temperature cycling protocol for the amplification of genes and mature miRNAs was 95 °C for 15 s, 56 °C for 30 s and 60 °C for 30 s for 40 reaction cycles, or 95 °C for 10 s, 60 °C for 20 s and 72 °C for 10 s for 40 reaction cycles, respectively. The housekeeping genes GAPDH were used as endogenous mRNA and the expression levels of miRNAs were normalized against U6. The results were calculated using the 2^−ΔCt^ method. Detailed information on the primer pairs is shown in Table 2.

**Table 2.** Primers

Gene	Primer sequence (5′ - 3′)
*GAPDH*	
Forward	GGACCTGACCTGCCGTCTAG
Reverse	GTAGCCCAGGATGCCCTTGA
*Pten*	
Forward	CACCAGTTCGTCCCTTTCCAG
Reverse	AAGACCATAACCCACCACAGC
*BCL6*	
Forward	TTCCGCTACAAGGGCAAC
Reverse	TGCAACGATAGGGTTTCTCA
HsnRNA U6 primer	HmiRQP9001 (GeneCopoeia)
hsa-miR-155-5p	HmiRQP0221 (GeneCopoeia)
hsa-miR-146a-5p	HmiRQP0196 (GeneCopoeia)
hsa-miR-17-5p	HmiRQP0230 (GeneCopoeia)
hsa-miR-18a-5p	HmiRQP0255 (GeneCopoeia)
hsa-miR-19a-3p	HmiRQP0293 (GeneCopoeia)
hsa-miR-19b-1-5p	HmiRQP0294 (GeneCopoeia)
hsa-miR-20a-5p	HmiRQP0312 (GeneCopoeia)
hsa-miR-92a-1-5p	HmiRQP0831 (GeneCopoeia)
hsa-miR-339-5p	HmiRQP0427 (GeneCopoeia)
hsa-miR-10a-5p	HmiRQP0032 (GeneCopoeia)

### Statistical analysis

For comparison between two continuous populations, two-tailed Student’s *t* tests and a Wilcoxon signed rank test were carried out as appropriate. Correlations between variables were performed using Pearson’s or Spearman correlation coefficients as appropriate. We used SPSS 15.0 software (SPSS Inc., Chicago, IL, USA) and GraphPad Prism 5 software (GraphPad Software Inc., La Jolla, CA) to perform statistical analyses. *P* values < 0.05 were considered significant. (*, *P* < 0.05; **, *P* < 0.01; ***, *P* < 0.001).

## Result

### Low responders to the hepatitis B vaccine presented lower frequencies of total and cTfh cells than hyper responders

**Fig. 2:**
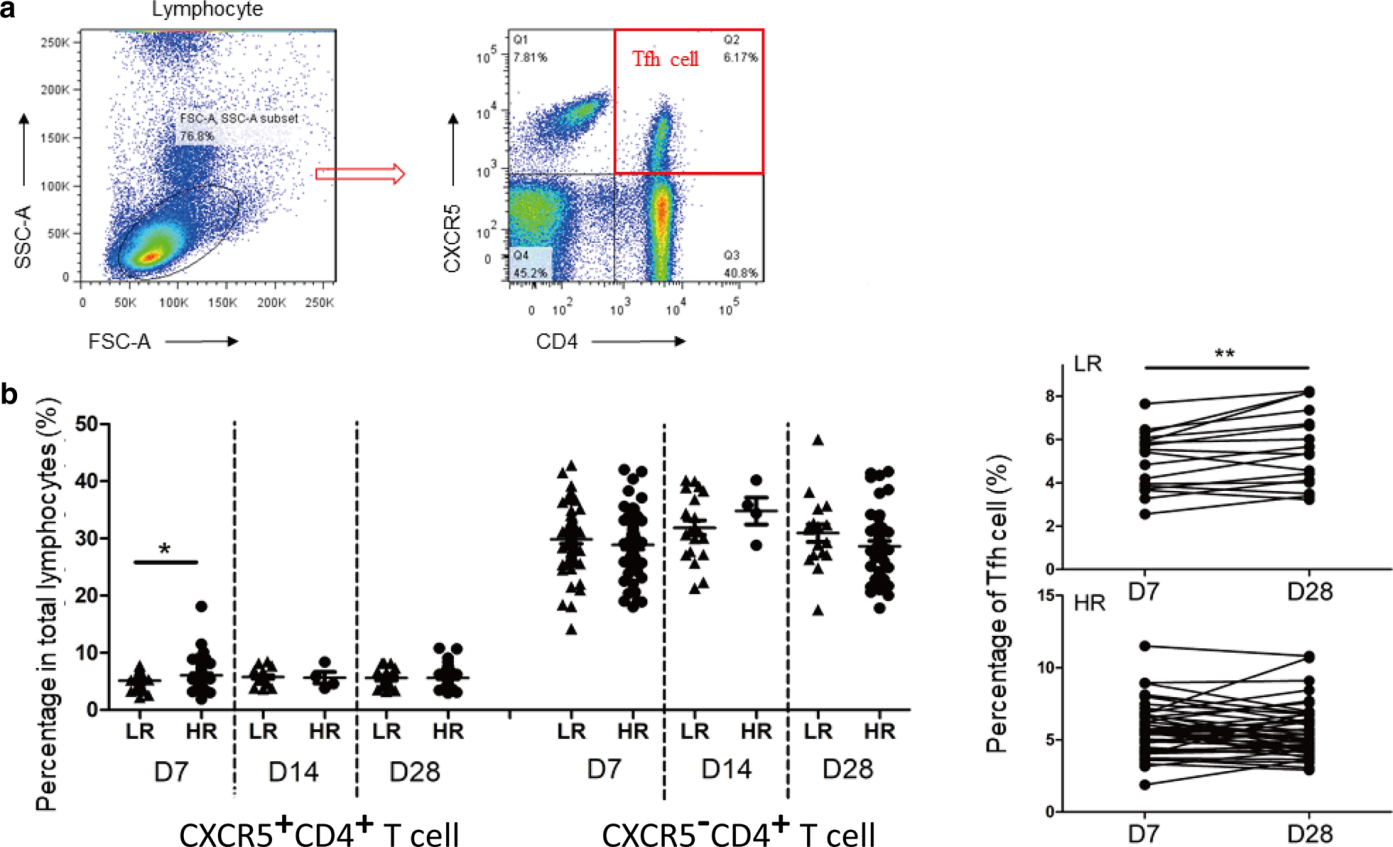
Low responder to the hepatitis B vaccine presented lower frequencies of cTfh cells than controls. **a** Representative dot plots of gating strategy. **b** Comparison frequencies of CXCR5^+^CD4^+^T cell, CXCR5^−^CD4^+^T cell between LR and HR at D7, D14 and D28 and kinetics of Tfh cell in LR and HR from D7 to D28. Median and range bars represent mean ± SEMs *LR* low responders, *HR* hyper responders, *D7* range 7–8 days after vaccination, *D14* range 14–15 days after vaccination, *D28* range 28–29 days after vaccination

The gating strategy is shown in Fig. 2a. We found the cTfh cells from low-responsive groups (n = 61) presented lower frequencies than the hyper-responsive groups (n = 56) at D7 and the frequency profiles were remarkably different between D7 and D28 (Fig. 2b). Of note, the frequency of CXCR5^˗^CD4^+^ T cells did not change between LR and HR at different time points.

### A skewed cTfh population away from cTfh2 and cTfh17 toward cTfh1 in the low responders to hepatitis B vaccine

**Fig. 3:**
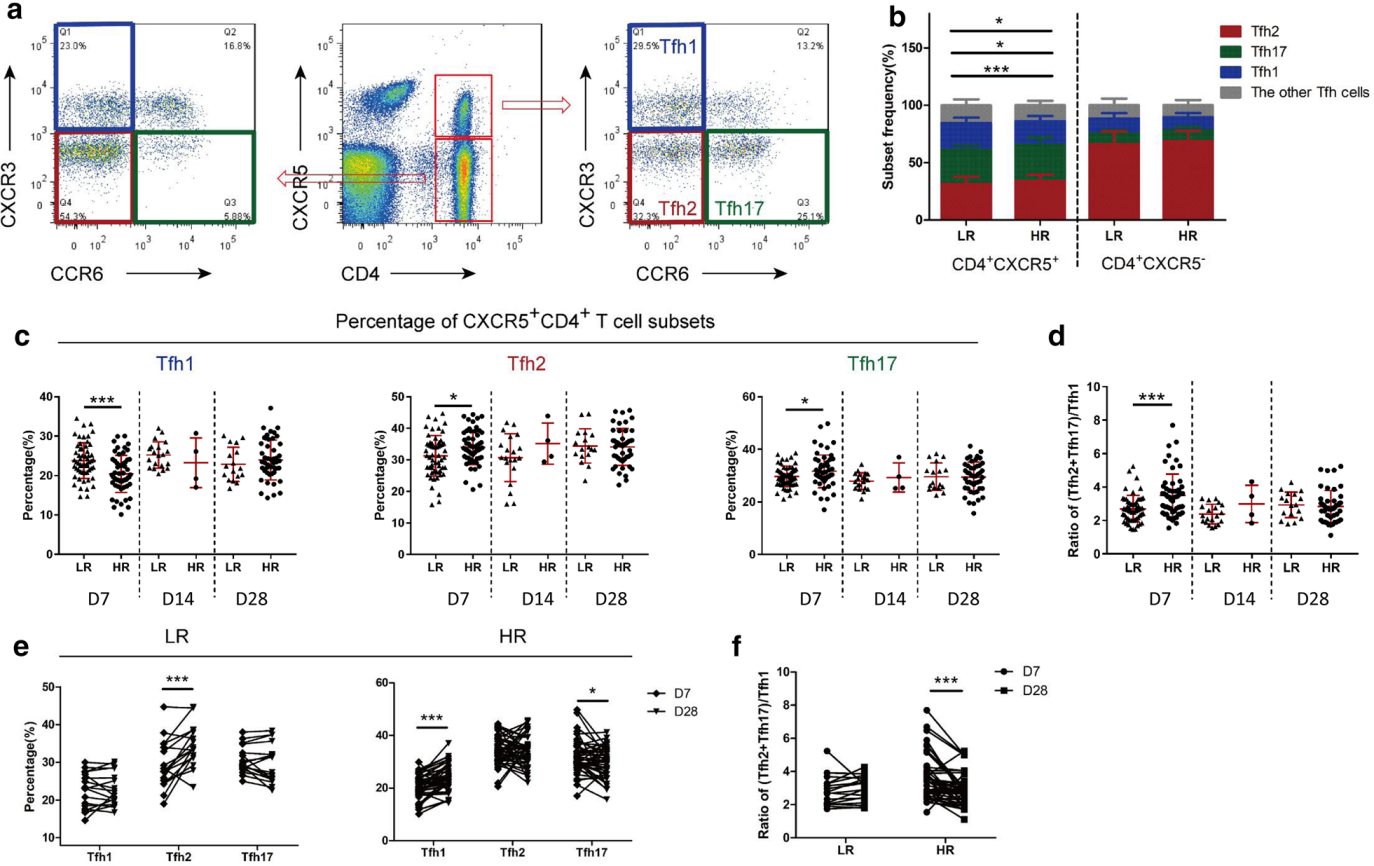
A skewed cTfh population away from cTfh2 and cTfh17 toward cTfh1 in low responder to the hepatitis B vaccine. **a** Representative dot plots of gating strategy for the analysis of CCR6 and CXCR3 expression in CXCR5^+^CD4^+^T cell or CXCR5^−^CD4^+^T cell population. **b** Subset frequency in CXCR5^+^CD4^+^T cell or CXCR5^−^CD4^+^T cell population between LR and HR at D7. **c** Comparison frequencies of Tfh1 cell, Tfh2 cell, Tfh17 cell between LR and HR at D7, D14 and D28. **d** Comparison ratio of (Tfh2 + Tfh17)/Tfh1 cell between LR and HR at D7, D14 and D28. **e** Kinetics of Tfh1 cell, Tfh2 cell and Tfh17 cell in LR and HR from D7 to D28. **f** Kinetics of ratio of (Tfh2 + Tfh17)/Tfh1 cell in LR and HR from D7 to D28. Median and range bars represent mean ± SD *LR* low responders, *HR* hyper responders, *D7* range 7–8 days after vaccination, *D14* range 14–15 days after vaccination, *D28* range 28–29 days after vaccination

Given the growing recognition of the different capacities of Tfh cell subsets in supporting antibody secretion, we further explore whether distribution of specialized cTfh subsets contributes to the low responsiveness to hepatitis B vaccine. The gating strategy is shown in Fig. 3a. As illustrated in Fig. 3b, cTfh cell subsets showed a significantly skewed distribution between the two groups at D7. Specifically, this phenomenon did not appear in CXCR5^˗^CD4^+^ T cell subsets (Fig. 3b). Regarding the different time points, the pool of CXCR5^+^CD4^+^ T cells from the LR contained fewer cTfh2 cells and cTfh17 cells but more cTfh1 cells compared with the HR at D7 (Fig. 3c). For convenience, the ratio of (Tfh2 + Tfh17)/Tfh1 was used to show the skewed distribution of the subsets and significance was observed in the ratio as well (Fig. 3d). The kinetics of changes in the subsets also demonstrated an increase of cTfh2 in the LR from D7 to D28 and an increase of cTfh1 and a decrease of cTfh17 in the HR from D7 to D28 (Fig. 3e). A decrease in the ratio of (Tfh2 + Tfh17)/Tfh1 was also observed in the HR.

### An imbalance in the different subsets of B cells emerged in the low responders to hepatitis B vaccine

**Fig. 4:**
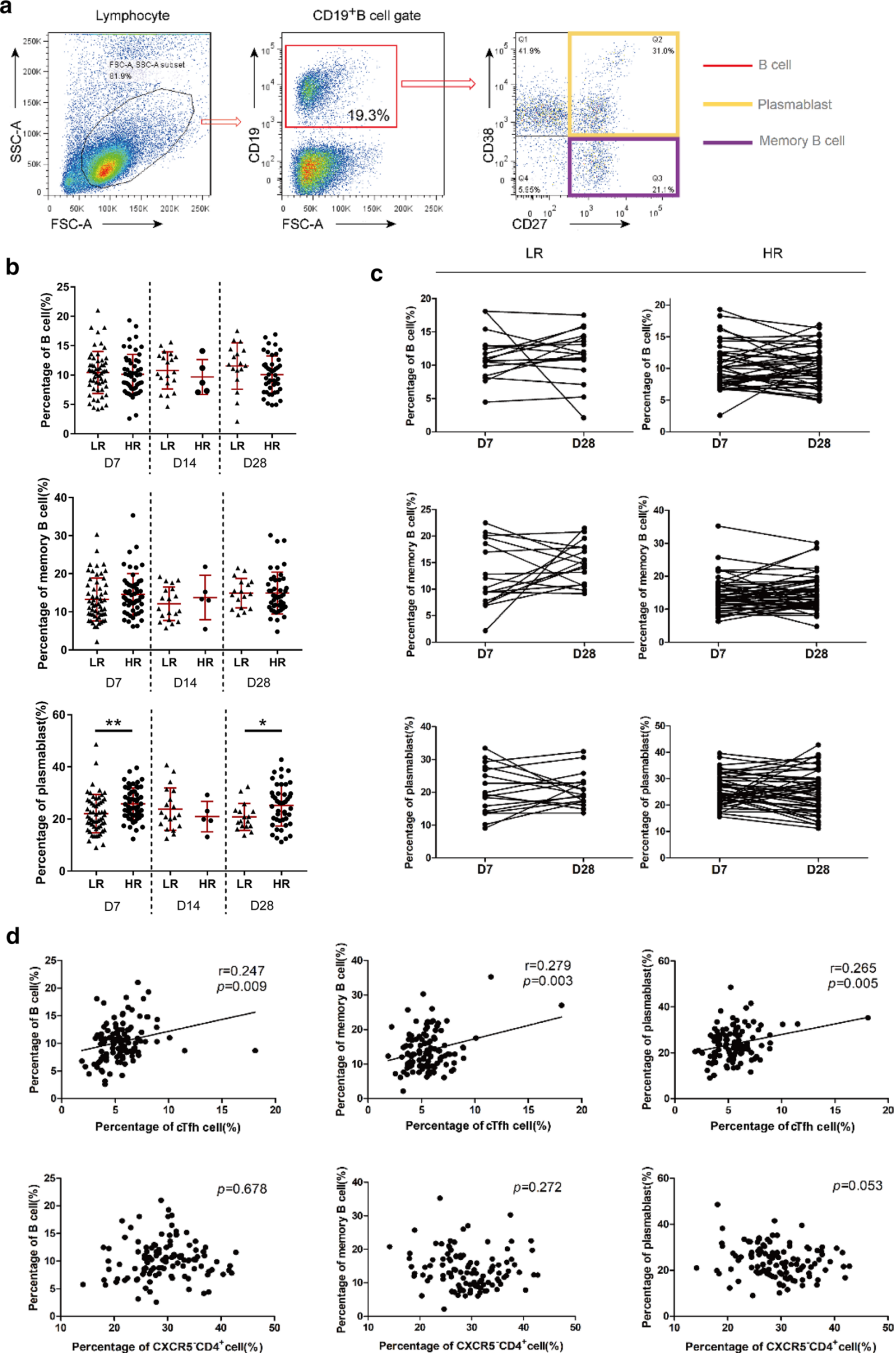
An imbalance of different subsets of B cells emerge in low responder to the hepatitis B vaccine. **a** Representative dot plots of gating strategy for the analysis of CD27 and CD38 expression in CD19^+^B cell. **b** Comparison frequencies of B cell, memory B cell and plasmablasts cell between LR and HR at D7, D14 and D28. **c** Kinetics of B cell, memory B cell and plasmablasts cell in LR and HR from D7 to D28. **d** Correlation between frequencies of cTfh cells or CXCR5^−^CD4^+^T cell and B cell subset at D7. Median and range bars represent mean ± SD *LR* low responders, *HR* hyper responders, *D7* range 7–8 days after vaccination, *D14* range 14–15 days after vaccination, *D28* range 28–29 days after vaccination

Despite an increased understanding of the role of Tfh cells, little was still known regarding the skewing of B cell compartments in attenuating hepatitis B antibody responses. CD27^+^CD38^+^CD19^+^ B cells and CD27^+^CD38^˗^CD19^+^B cells represent antibody-secreting plasma cells and activated memory B cells, respectively (Wrammert et al. 2008). The gating strategy for analyzing CD19^+^ B cell subsets is shown in Fig. 4a. Similar results were observed for both B cells and memory B cells in that there was no significant difference between the two groups at D7, D14 and D28 (Fig. 4b). However, plasmablasts were found to increase in HR compared with LR both at D7 and D28 (Fig. 4c). In addition, the increase in cTfh cells positively correlated with the increased frequency of B cells, memory B cells and plasmablasts. Although there was a correlation between CXCR5^˗^CD4^+^ T cells and B cell subsets, this did not reach statistical significance (Fig. 4d).

### Emergence of cTfh cell subsets was associated with increased levels of IL-21 and anti-HBs

**Fig. 5:**
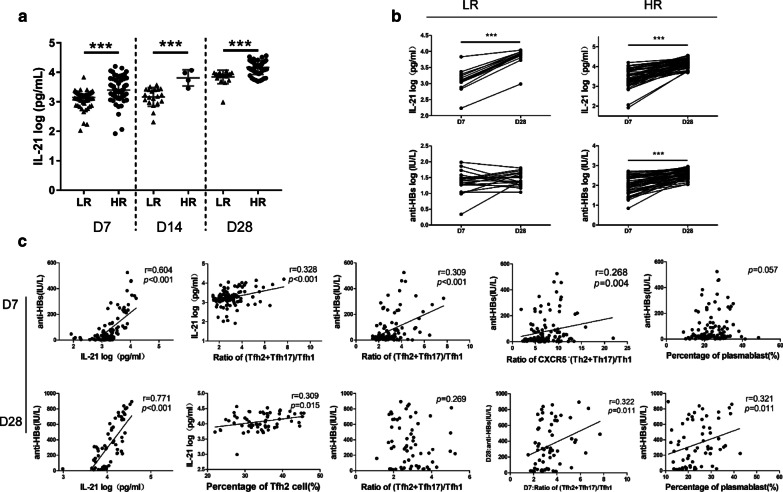
cTfh cells was associated with increased level of IL-21 and anti-HBs. **a** Comparison frequencies of IL-21 between LR and HR at D7, D14 and D28. **b** Kinetics of IL-21 and anti-HBs in LR and HR from D7 to D28. **c** Correlation analysis at D7 and D28. Median and range bars represent mean ± SD or median with interquartile range as appropriate *LR* low responders, *HR* hyper responders, *D7* range 7–8 days after vaccination, *D14* range 14–15 days after vaccination, *D28* range 28–29 days after vaccination

In cytokine profiling, IL-21 is highly expressed in Tfh cells and potently accelerated the development of plasmablasts (Crotty 2011). Considering that Tfh cells are the main producers of IL-21 in serum (Crotty 2011), we detected IL-21 expression. The results showed compared with responders, IL-21 was significantly decreased in LR at all three time points (Fig.5a). Dynamic changes showed IL-21 levels increased greatly from D7 to D28 in both the LR and HR (Fig. 5b). There was a strong upward tendency observed for anti-HBs in the HR, but the trend was not significant in the LR, which was consistent with the poor immune response (Fig. 5b).

We then questioned whether the emergence of IL-21 correlated with the generation of protective antibody responses. The results showed a striking correlation between IL-21 and anti-HBs at both D7 and D28 and the (Tfh2 + Tfh17)/Tfh1 ratio was also positively related to IL-21 expression at D7. Given that Tfh2 and Tfh17 cells could secrete IL-21 to efficiently assist antibody generation (Morita et al. 2011), we also determined whether the frequency of Tfh2 and Tfh17 cells was associated with IL-21. As assumed, there was a correlation between the percentage of cTfh2 cells and IL-21 at D28. Indeed, antibody titers correlated with the (Tfh2 + Tfh17)/Tfh1 ratio only at D7 and unexpectedly with the ratio of CXCR5^˗^(Th2 + Th17)/Th1. Considering a later antibody response, we determined the correlation between the (Tfh2 + Tfh17)/Tfh1 ratio at D7 and antibody responses at D28 and found weak associations, implying cTfh cells might predict vaccine-induced antibody responses. After further analysis, an expected correlation was found between plasmablasts and anti-HBs at D28, but this correlation was not significant at D7 (Fig. 5c).

### MiR-19b-1 and miR-92a-1 correlated with the distribution of cTfh cell subsets and antibody production

Understanding the immunological mechanism regulating Tfh cell process deeply may facilitate the development of strategies to precisely improve poor vaccine responses. It has previously been reported that the critical microRNAs involved in Tfh cell differentiation are contained miR-17 ~ 92 cluster (including miR-17, miR-18a, miR-19a, miR-19b-1, miR-20a, miR-92a-1), miR-146 and miR-155 (Maul and Baumjohann 2016). Additionally, BCL6 was also reported to be an indispensable transcription factor and our previous findings revealed significant dynamic changes in BCL6 in response to the hepatitis B vaccine. To investigate further, StarBase v3.0 (http://starbase.sysu.edu.cn/index.php) (Li et al. 2014) was used to predict candidate miRNAs targeting BCL6. We screened the top 12 predicted miRNAs according to strict parameters predicted by four programs simultaneously (Additional file1: Fig. S1a). Among the predicted miRNAs, hsa-miR-10a-5p and hsa-miR-339a-5p was selected for further study, which were found to be involved in the immune system according to the literature, indicating their potential role in contributing to vaccine-induced antibody levels. The putative binding sites of the two miRNAs are shown in Additional file 1: Fig. S1a. However, further studies are needed to validate whether by targeting BCL6, miR-10a-5p and miR-339a-5p are involved in the low-responsiveness to hepatitis B vaccine.

**Fig. 6:**
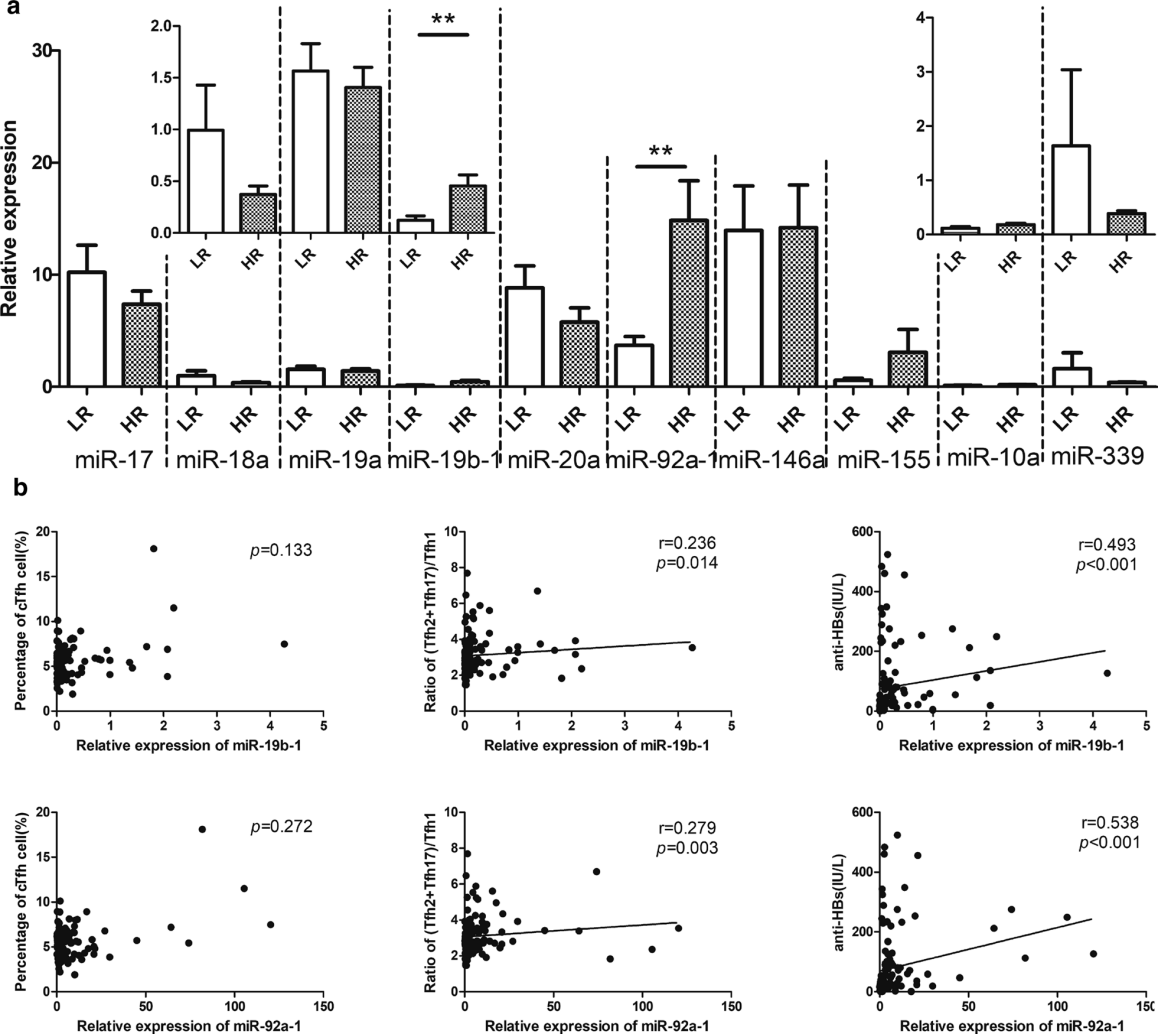
MiR-19b-1 and miR-92a-1 correlated with cTfh cell subsets distribution and antibody production. **a** Comparison of miRNAs relative expression by qRT-PCR range 7–8 days after vaccination. **b** Correlation between miR-19b-1, miR-92a-1 and cTfh cell, ratio of (Tfh2 + Tfh17)/Tfh1 cell, anti-HBs range 7–8 days after vaccination *LR* low responders, *HR* hyper responders

Unexpectedly, Bcl-6 mRNA expression levels in LR were similar to those in HR (data not shown). And predicted hsa-miR-10a-5p and hsa-miR-339a-5p mRNA expression levels in LR were also similar to those in HR. Among reported significant changes in the relative expression were observed in miR-19b-1 and miR-92a-1 at D7 (Fig. 6a) and there was a upward trend observed with miR-19b-1 and miR-92a-1 from D7 to D28 in the HR (Additional file 1: Fig. S1b). The HR displayed a higher level of PTEN, encoded by a target gene of the miR-17 ~ 92 cluster at D28 (Maul and Baumjohann 2016). (Additional file1: Fig. S1c). Lastly, both miR-19b-1 and miR-92a-1 were also positively associated with the ratio of (Tfh2 + Tfh17)/Tfh1 and anti-HBs but not cTfh cells (Fig. 6b).

## Discussion

HBV infection leads to a high risk of death from cirrhosis and hepatocellular carcinoma; however, there has been an overall decline in infection rates due to the currently available safe and effective hepatitis B vaccine and standard vaccination schedules (WHO 2017). Despite this, 5–10 % of individuals do not exhibit an antibody response, thereby limiting hepatitis B vaccine efficacy. To our best knowledge, our study is the first to report the specific and precise contribution of Tfh cells subsets and miRNA regulatory mechanisms to the low-responsiveness to hepatitis B vaccination. Our findings provided evidence of promising targets with which to improve vaccine efficacy against HBV.

Based on large-scale screening, we explored the specific cellular mechanisms involved in the low response to hepatitis B vaccination among healthy volunteers. The low response to hepatitis B vaccine presented lower frequencies of cTfh cells, but not CXCR5^−^CD4^+^ T cells, than HR, indicating that cTfh cells rather than other CD4^+^T cell subsets were a better marker for poor responses to hepatitis B vaccination. Such a result was consistent with previous data showing insufficient cTfh cells among older people with a poor antibody response following influenza vaccination (Herati et al. 2014). Moreover, we reported a significant difference in the cTfh subset in different groups and the association between the serum anti-HBs levels and three cell subsets, as well as a skew in the cTfh subset emphasizing the critical support of this subset for a strong antibody response. This result indicated the low hepatitis B antibody response might also be dependent on the skew of the cTfh subset and the increase in Tfh cell number alone may not be sufficient to improve the antibody response in line with similar findings regarding Tfh cell numbers in mice (Deenick et al. 2010). In addition, Tfh2 and Tfh17 cells emerged as the subsets with superior capacity than others in cTfh cell pools to facilitate B cell differentiation and maturation. We assumed that the presence of preponderant Tfh2 and Tfh17 cells in HR favored strong protective antibody responses. There might be synergistic effects between Tfh2 cells and Tfh17 cells in inducing an anti-HBs response, but which subset plays a dominant role needs to be further studied. The phenomenon of the skew in the cTfh population away from cTfh2 and cTfh17 both toward cTfh1 in LR to the hepatitis B vaccine implied a decrease in functional cell subsets leading to the induction of poor antibody responses. This decrease in the percentage of cTfh1 cells among the total Tfh cell pool was partially due to an increase in the marked predominance of cTfh2 and cTfh17 subsets. Similar results showed both Tfh2 and Tfh17 cells correlated with the development of broadly neutralizing antibodies to HIV (Locci et al. 2013). According to our findings, hepatitis B vaccination induced a specific change in the ratio of (Tfh2 + Tfh17)/Tfh1 cells and the predominance of cTfh2 or cTfh17 subsets was also seen in response to other vaccines. PD-1^+^ICOS^+^Tfh2 cells were induced after human papillomavirus vaccination (Matsui et al. 2015)and the Tfh17 cell subset was induced during the antibody response to rVSV-ZEBOV Ebola vaccine (Farooq et al. 2016). By contrast, the Tfh1 subset dominated in response to influenza vaccine, implying suboptimal antibody responses to influenza (Bentebibel et al. 2013; Bentebibel et al. 2016). Based on these findings, we assumed that the low antibody response to hepatitis B vaccine was at least partially attributed to profound skewing of the functional subsets of cells, and was not simply a result of the total number of cTfh cells in LR.

Tfh cells are essential for B cells subset production such as plasmablasts and memory B cells (Farooq et al. 2016; Bentebibel et al. 2016; Bekele et al. 2017). Our study established correlations between plasmablasts and cTfh cells and antibody levels, which indirectly demonstrated the role of cTfh cells in LR to the hepatitis B vaccine. Notably, a significant difference was only observed in plasmablasts and a positive correlation was established between plasmablasts and cTfh cells, suggesting the possibility that Tfh cells are defective in the function of helping B cells differentiate into antibody-producing plasmablasts in LR. Furthermore, we analyzed the relationship between the B cell subset and CXCR5^˗^CD4^+^ T cells, but no correlation was evident indicating Tfh cells rather than other CD4^+^T cell subsets may be responsible for B cell differentiation and function in LR. In addition, IL-21 secreted from Tfh cells was of importance for GC B cell differentiation and maturation (Berglund et al. 2013), in accordance with the higher frequency of plasmablasts in HR. Our data indicated a gradual and striking increase in serum IL-21 expression both in LR and HR over time and suggested IL-21 to be a dominant cytokine in anti-HBs production, which was in agreement with preponderant Tfh2 and Tfh17 cell subsets, which are the main Tfh cells secreting IL-21 and a cell type that is decreased in LR.

The defined contribution of cTfh cells in low-responsiveness to the hepatitis B vaccine provided insight into the regulatory mechanisms in this process which may facilitate the development of strategies to precisely improve poor vaccine responses. Over recent years, increasing evidence has shown miRNAs have specific functions in T cell differentiation and plasticity (Maul and Baumjohann 2016). Expression of the miR-17 ~ 92 cluster has been shown in immune diseases (Wu et al. 2018), but not widely observed in response to vaccination. Transcriptional profiles suggest that low-responsiveness to the hepatitis B vaccine was related to the role of miR-19b-1 and miR-92a-1. Interestingly, some reports have suggested the miR-17 ~ 92 cluster plays an opposite role during the differentiation of Tfh cells (Yu et al. 2009; Baumjohann et al. 2013), whereas our results showed positive regulation association of miR-17 ~ 92 in Tfh cell differentiation. According to our result of decreased levels of the target gene of PTEN in HR, miR-17 ~ 92 might inhibit PTEN. However, whether repression of PTEN by miR-19b-1 and miR-92a-1 positively regulates Tfh cell differentiation needed to be studied further. We did not observe decreased levels of Bcl-6 in LR, which was consistent with previous reports in other immunological diseases (Morita et al. 2011; Zhang et al. 2016)and the fact that cTfh cells in blood failed to express the same levels of Bcl-6 as lymphoid Tfh cells (Schmitt et al. 2014).

Our study has some limitations. The samples in our study is limited. The differences between LR and HR may depend on just small number of individuals. And our finding limited to association rather than causation between biomarkers and low-responsiveness to hepatitis B vaccination. Large sample cohort study should be performed to confirm our findings furtherly. We dynamically analyzed the immunological parameters rather than focusing on a stationary time point like previous research. However, there was insufficient data at D14 and D28 compared with D7. However, the D7 time point, at which the main immunological events in the low response to hepatitis B vaccination occurred, had been fully defined. In addition, it is obviously a challenge to obtain 20 mL blood sampling from children. As a result, we recruited eligible volunteers limited to young adults. Whether our findings are fully applicable to children with vaccination failure needs to be further investigated.

## Conclusions

In the current study, we identified specific changes in Tfh cells that were related to low hepatitis B vaccine-induced antibody generation. Strikingly, there was a skew toward highly functional cTfh2 and/or cTfh17 cell populations and away from Tfh1 cells and skewing of Tfh cell subsets which might produce the dominant cytokine IL-21 important for anti-HBs production. Furthermore, miR-19b-1 and miR-92a-1-modulated Tfh cells might potentially serve as targets with which to improve vaccine efficacy in LR to hepatitis B vaccine.

## Supplementary Information

**Additional file 1: Figure S1**. MiR-19b-1 and miR-92a-1 correlated with cTfh cell subsets distribution and antibody production.

## Data Availability

The datasets used and/or analysed during the current study are available from the corresponding author on reasonable request.

## Abbreviations

cTfh cell: Circulating follicular helper T cells
HBV: Hepatitis B virus
LR: Low responders
HR: Hyper responders
