# Supplementary figures and images for: Circulating Tfh cell and subsets distribution are associated with low‐responsiveness to hepatitis B vaccination
Source: Mol Med. 2021 Apr 1;27:32. doi: 10.1186/s10020-021-00290-7 (PMC8015036; doi:10.1186/s10020-021-00290-7)

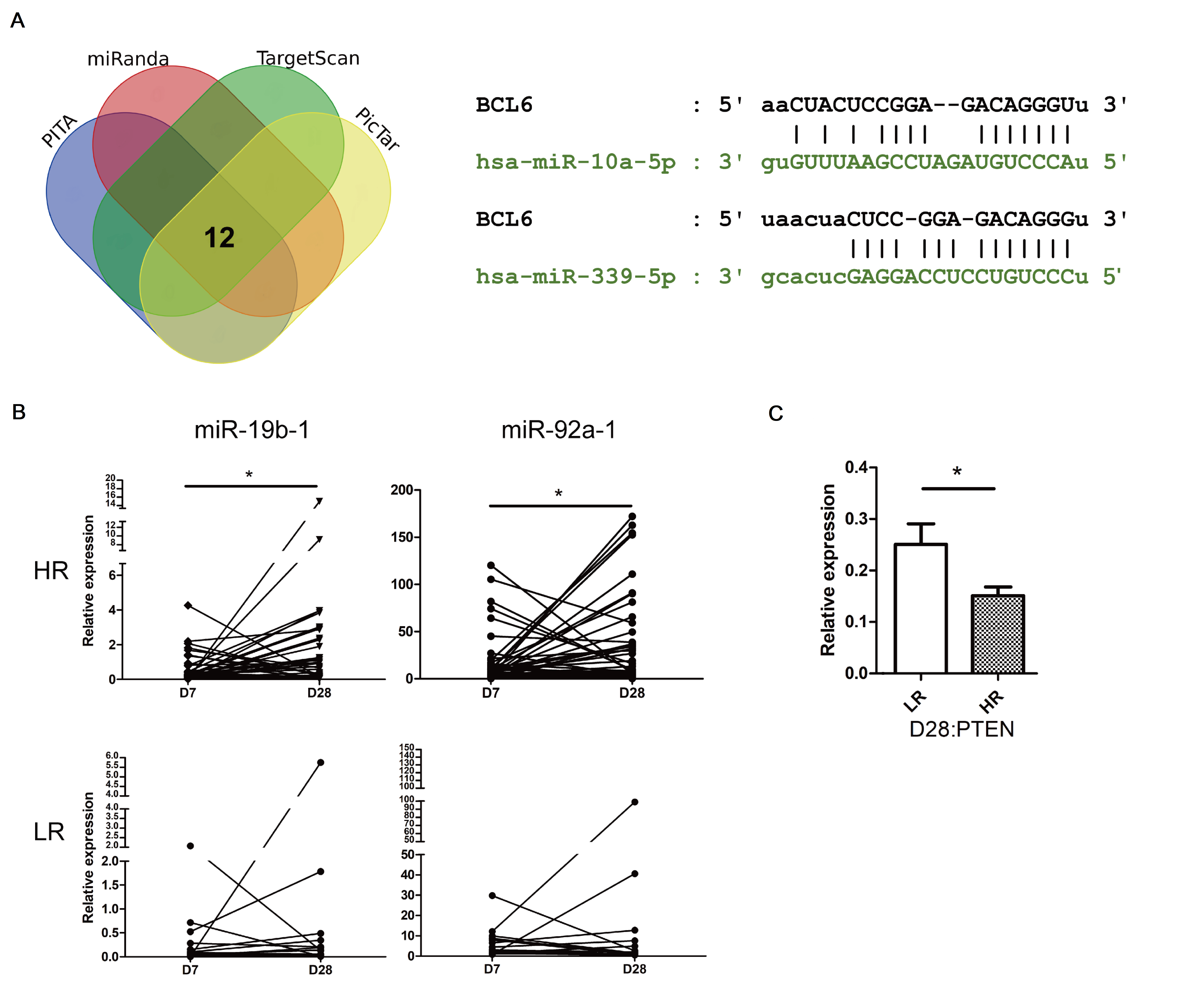

Supplement: Supplementary file 1 — Additional file 1: Figure S1. MiR-19b-1 and miR-92a-1 correlated with cTfh cell subsets distribution and antibody production. [file 10020_2021_290_MOESM1_ESM.tif]
